# Safety analysis of pemigatinib leveraging the US Food and Drug administration adverse event reporting system

**DOI:** 10.3389/fphar.2023.1194545

**Published:** 2023-07-24

**Authors:** Ying Zhang, Li Ran, Yongchao Liang, Yanqiu Zhang, Zhuoling An

**Affiliations:** Department of Pharmacy, Beijing Chao-Yang Hospital, Capital Medical University, Beijing, China

**Keywords:** pemigatinib, Food and Drug Administration adverse event reporting system, disproportionality analysis, real-word study, Advers drug events

## Abstract

**Background:** Cholangiocarcinoma (CCA) is a highly lethal and aggressive epithelial tumor of the hepatobiliary system. A poor prognosis, propensity for relapse, low chance of cure and survival are some of its hallmarks. Pemigatinib, the first targeted treatment for CCA in the United States, has been demonstrated to have a significant response rate and encouraging survival data in early-phase trials. The adverse events (AEs) of pemigatinib must also be determined.

**Objective:** To understand more deeply the safety of pemigatinib in the real world through data-mining of the US Food and Drug Administration (FDA) Adverse Event Reporting System (FAERS).

**Methods:** Disproportionality analysis was employed in a retrospective pharmacovigilance investigation to identify the AEs linked to pemigatinib use as signals. Data were collected between 1 January 2020 to 30 June 2022. Four data-mining methods (proportional reporting odds ratio; proportional reporting ratio; Bayesian confidence propagation neural networks of information components; empirical Bayes geometric means) were used to calculate disproportionality.

**Results:** A total of 203 cases using pemigatinib as the prime-suspect medication were found in our search, which involved 99 preferred terms (PTs). Thirteen signals of pemigatinib-induced AEs in seven System Organ Classes were detected after confirming the four algorithms simultaneously. Nephrolithiasis was an unexpected significant AE not listed on the drug label found in our data-mining. Comparison of the differences between pemigatinib and platinum drugs in terms of 33 PTs revealed that 13 PTs also met the criteria of the four algorithms. Ten of these PTs were identical to those compared with all other drugs, in which (excluding a reduction in phosphorus in blood) other PT signal values were higher than those of all other drugs tested. However, comparison of the differences between pemigatinib and infigratinib in terms of the 33 PTs revealed no significant signals in each algorithm method.

**Conclusion:** Some significant signals were detected between pemigatinib use and AEs. PTs with apparently strong signals and PTs not mentioned in the label should be taken seriously.

## Introduction

Cholangiocarcinoma (CCA) is an extremely deadly and aggressive epithelial tumor of the hepatobiliary system that can be classified as “intrahepatic,” “perihilar,” or “distal” based on its origin (Brindley et al., 2021). CCA can be associated with chronic inflammation of the biliary tract attributable to choledocholithiasis, cholelithiasis, or primary sclerosing cholangitis, but most CCAs go undiagnosed. The incidence of CCA is increasing, and it carries a poor prognosis, particularly intrahepatic CCA (Bridgewater et al., 2014; Goyal et al., 2021; Kam et al., 2021).

Some patients with CCA qualify for potentially curative surgical procedures, including resection or liver transplantation (Brindley et al., 2021; National Comprehensive Cancer Network, 2021). For individuals with advanced-stage CCA who are ineligible for surgical or locoregional treatments, first-line chemotherapy with cisplatin and gemcitabine is an option (Brindley et al., 2021).

CCA has a poor prognosis because most patients are diagnosed with advanced disease, resulting in poor responses to systemic therapies (e.g., palliative chemotherapy), making it harder to achieve complete remission (Wu and Yeh, 2021). The median duration of survival from CCA is shorter than 2 years, and 90% of individuals die within 5 years of the initial diagnosis (Simile et al., 2019; American Cancer Society, 2020).

Resection can eradicate early-stage CCA entirely, but most patients experience recurrence within 2 years. The short median survival of this disease highlights its aggressive nature and the need for optimized treatments (Goyal et al., 2021; Cheng et al., 2022).

An increasing number of targeted therapies exhibit positive clinical activity, and the treatment approach for advanced CCA continues to evolve (Kam et al., 2021). Fibroblast growth factor receptor 2 (FGFR2) fusions, which drive the pathogenesis of intrahepatic CCA, can be targeted therapeutically (Goyal et al., 2021).

**FIGURE 1:**
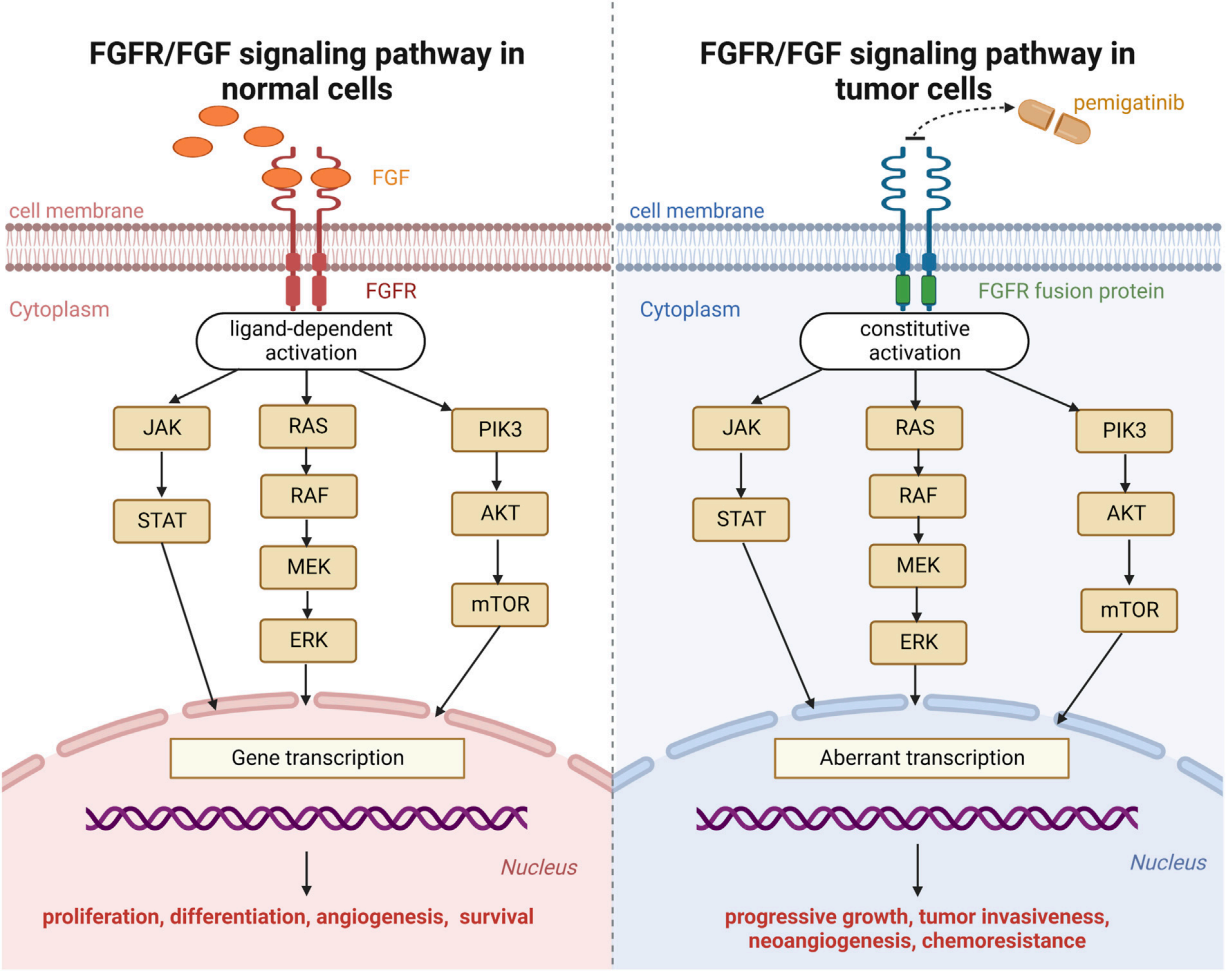
Fibroblast growth factor receptor (FGFR) signaling pathway in normal cells and tumor cells.

The primary components of the FGFR signaling pathway in human cells are FGFR 1–4 transmembrane receptor proteins with intracellular tyrosine-kinase domains and 23 FGF ligands (Kommalapati et al., 2021). As soon as FGF activates these receptors, this link causes FGFRs to dimerize. This action triggers autophosphorylation of the intracellular kinase domain and activation of downstream pathways, resulting in the proliferation, differentiation, angiogenesis, and survival of normal cells (Figure 1) (Costa et al., 2016; Facchinetti et al., 2020).

Pemigatinib is a small-molecule inhibitor of FGFR1, FGFR2, and FGFR3. Upon binding with the kinase domain of FGFR fusion proteins, pemigatinib inhibits downstream oncogenic signaling pathways (Janus kinase-signal transducer and activator of transcription; mitogen-activated protein kinase; phosphatidylinositol 3-kinase-protein kinase B-mammalian target of rapamycin). The mechanism of action of pemigatinib on neoplastic cells is shown in Figure 1 (Chakrabarti et al., 2022).

In April 2020 in the USA, pemigatinib received accelerated approval for the treatment of adults with previously treated, unresectable, locally advanced, or metastatic CCA carrying an FGFR2 fusion or other rearrangement as determined by Food and Drug Administration (FDA)-approved test. It was the first targeted treatment for CCA approved in the United States, and was linked to an excellent response and positive survival data (Hoy, 2020). Pemigatinib produced an objective response rate of 35.5% in a phase-II study of patients with CCA and *FGFR2* fusions/rearrangements (Abou-Alfa et al., 2020).

In view of the remarkable efficacy and widespread application of pemigatinib in CCA treatment, being aware of its adverse reactions (AEs) is important. To provide a comprehensive and valuable reference for the safety of pemigatinib in the real world, we undertook a disproportionality analysis using a sizable pharmacovigilance database to characterize and evaluate pemigatinib-related AEs.

## Materials and methods

### Data sources and study variables

A retrospective study was conducted using the FDA Adverse Event Reporting System (FAERS). The FAERS is a database. The data mined in the present study were taken from the FAERS *via* OpenVigil covering the period 1 January 2020 to 30 June 2022.

The FAERS includes reports on AEs, medication errors, and quality issues of products resulting in AEs that are submitted to the FDA. The FAERS assists the post-marketing safety surveillance program for pharmaceutical and therapeutic biologic products for the FDA (U.S. Food and Drug Administration, 2022a). The FDA releases data to the public and allows pharmacoepidemiological investigations and/or pharmacovigilance evaluations (Sakaeda et al., 2013).

Using data from the FAERS, the Internet-based pharmacovigilance analytical tool OpenVigil FDA can access domestic and international pharmacovigilance data. As the first publicly accessible tool, OpenVigil FDA applies pharmacovigilance data to real-life clinical issues (Böhm et al., 2016; Meng et al., 2021; Papazisis et al., 2021).

### Procedures for pharmacovigilance study

The brand name “PEMAZYRE” and generic name “pemigatinib” were used to identify related records because the FAERS does not employ a standard classification system for pharmaceutical agents. According to FDA instructions, a de-duplication step was undertaken before statistical analyses by choosing the most recent version when the case ID was identical, which reduced the number of reports to 203 (Shu et al., 2022). The most up-to-date information about a case was contained in its most recent version (U.S. Food and Drug Administration, 2022b).

To improve accuracy, the role code of AEs was included only as the primary-suspect drug. In the FAERS, each report is coded using preferred terms (PTs) from Medical Dictionary for Regulatory Activities (MedDRA) version 24.0. All AE reports regarding pemigatinib in the FAERS were identified to describe the frequency and signal strength based on MedDRA at System Organ Class (SOC) and PT levels. Only PTs with ≥3 reports were used for signal detection in our study.

### Data-mining and statistical analyses

In the context of pharmacovigilance, disproportionality analysis involves evaluation of the proportion of AEs occurring between a given agent and all other pharmaceutical agents. Disproportionality analysis is a fundamental analytical method in pharmacovigilance studies for detecting drug-associated AEs as “signals” (Hu et al., 2020). The general principle is that a signal is considered to have been generated in the data-extraction period if the prevalence of a specific AE associated with a specific drug is significantly higher than the background frequency in the FAERS and it reaches a certain threshold or criterion. Four data-mining methods were used to calculate disproportionality: proportional reporting odds ratio (ROR); proportional reporting ratio (PRR); Bayesian confidence propagation neural networks of information component (IC); empirical Bayes geometric mean (EBGM) (Hou et al., 2014; Ang et al., 2016).

Despite the lack of a “gold standard,” each data-mining method has advantages and disadvantages with respect to applicability in different situations and the possibility for implementation. Sensitivity, specificity, positive predictive value, and negative predictive value were calculated to evaluate the characteristics of signal-detection methods (Table 1) (Harvey et al., 2004; Kubota et al., 2004; Li et al., 2015). The use of multiple approaches is advised for AE surveillance using drugs rather than relying on only one approach.

**TABLE 1:** Sensitivity, specificity, positive and negative predictive values in signal detection methods.

Algorithm	Sensitivity	Specificity	PPV	NPV	Stability	Operability	Application	Consistency*(Kappa)
PRR^#^	1.00	0.76	0.83	1.00	low	Simple	British Adverse reaction Monitoring System (used)	ROR&PRR^#^ (kappa = 0.983)
ROR	1.00	0.77	0.83	1.00	lower	Simple	Netherlands Pharmacovigilance Centre Lareb	
MHRA	0.98	0.85	0.88	0.97	higher	Simple	MHRA EU	MHRA&BCPNN (kappa = 0.919)
BCPNN	1.00	1.00	1.00	1.00	high	Complex	WHO UMC	
MGPS	0.56	1.00	1.00	0.66	high	Complex	FDA	GPS&MHRA/BCPNN (kappa≈0.6)

PPV, positive predictive value; NPV, negative predictive values; MHRA, medicines and healthcare products regulatory agency; WHO UMC, world health organization uppsala monitoring centre.

*Otherwise, the values of kappa are less than 0.2.

#PRR, criteria is PRR≥2, N ≥ 3, no χ^2^.

To avoid bias in signal-detection results and to ensure detection accuracy, AE signals were considered to have been found only if all four algorithm conditions were met simultaneously. The formulae employed for calculations and the criteria of the four algorithms in the disproportionality analysis are presented in Table 2. Excel™ (Microsoft, Redmond, WA, United States) was employed for all data processing and statistical analyses.

**TABLE 2:** Four algorithms used for signal detection.

Algorithms	Equation	Criteria
ROR	ROR = ad/b/c	lower limit of 95% CI > 1, N ≥ 3
	95%CI = e^ln(ROR)±1.96(1/a+1/b+1/c+1/d)^0.5^	
PRR(MHRA)	PRR = a(c + d)/c/(a+b)	PRR≥2, χ^2^ ≥ 4, N ≥ 3
	χ^2^ = [(ad-bc)^2^](a+b + c + d)/[(a+b)(c + d)(a+c)(b + d)]	
BCPNN	IC = log_2_[(cxy+γxy) γ/(C+γ)]	IC025 > 0
	95%CI = IC ± 2SD	
MGPS	EBGM = a(a+b + c + d)/(a+c)/(a+b)	EBGM05 > 2
	95%CI = e^ln(EBGM)±1.96(1/a+1/b+1/c+1/d)^0.5^	

Equation.

^a^ Number of target AEs, of pemigatinib alone.

^b^ Number of other AEs, of pemigatinib alone.

c Number of target AEs, of all drugs excluding pemigatinib

^d^ Number of other AEs, of all drugs excluding pemigatinib; CI, confidence interval; N, number of reports; IC025, lower limit of the 95% CI, of IC; EBGM05, lower limit of the 95% CI, of EBGM.

## Results

### Descriptive analyses

In total, 3,344,771 records were submitted to the FAERS during the study period, 203 of which were related to the AEs elicited after pemigatinib use. The clinical characteristics of patients with pemigatinib-induced AEs are described in Table 3.

**TABLE 3:** Clinical characteristics of patients with pemigatinib-induced AEs.

Characteristics	Variable	N	%
Number of events		203	100
Gender	Male	75	36.95
	Female	104	51.23
	Unknown	24	11.82
Age(years)	<60	20	9.85
	60≤ and <80	12	5.91
	≥80	3	1.48
	Unknown	168	82.76
Reporting year	2020	101	49.75
	2021	88	43.35
	2022(to 0502/Q2)	14	6.90
Reported countries	Austria	1	0.49
	Canada	3	1.48
	Germany	7	3.45
	France	2	0.99
	Portugal	1	0.49
	Turkey	1	0.49
	United states	187	92.12
	Country not specified	1	0.49
Drug treatment regimen	Monotherapy	200	98.52
	Combination	3	1.48
	Cisplatin	1	0.49
	Nivolumab	1	0.49
	Cisplatin + Paclitaxel	1	0.49
Indications (TOP five)	Cholangiocarcinoma	166	81.77
	Other neoplasm malignant	13	6.40
	Myeloid/lymphoid neoplasms	6	2.96
	Pancreatic carcinoma	5	2.46
	Hepatobiliary cancer	4	1.97
Serious outcome*	Death	36	17.73
	Life-threatening	7	3.45
	Hospitalization	113	55.67
	Disability	8	3.94
	Other serious medical events	25	12.32
	unknown	28	13.79

*One event can have multiple outcomes.

Among these 203 reports, women were more likely to be affected than men (51.23% vs. 36.95%). Most reports (82.76%) did not include information on patient age and, thus, further analysis by age group was not possible. Because the drug was first approved in the United States in 2020, most reports (92.12%) were submitted from the United States, followed by Germany (3.45%) and Canada (1.48%). Pemigatinib-related AEs have been reported to the FAERS each year since 2020. For 200 events, the drug was administered alone, whereas three events involved combination therapy (two cases involving a two-drug combination and one case involving a three-drug combination). The indications for pemigatinib were CCA (81.77%), other malignant neoplasms (6.40%), and myeloid/lymphoid neoplasms (2.96%). Hospitalization (55.67%) was the most frequently reported severe outcome. In total, 36 (17.73%) and 25 (12.32%) pemigatinib-induced AEs resulted in death or other serious medical events, respectively.

### Spectrum of AEs and signal values associated with pemigatinib

Signal strengths and reports of pemigatinib at the SOC level are described in Table 4. Statistical analyses revealed that pemigatinib-induced AEs involved 18 SOCs. The most common SOCs were “general disorders and administration site”, “surgical and medical procedures,” and “gastrointestinal disorders”.

**TABLE 4:** Pemigatinib signal strength at the SOC level in the FAERS database.

System organ class (SOC)	N (%)	ROR (95%two-sided CI)	PRR (χ2)	IC (IC025)	EBGM (EBGM05)
General Disorders and Administration Site Conditions	72 (24.91)	1.62 (1.22–2.16)	1.40 (11.13)	0.47 (0.08)	1.40 (1.05)
Surgical and Medical Procedures△	70 (24.22)	58.68 (43.92–78.395)	38.79 (2593.49)	4.655 (4.26)	38.69 (28.96)
Gastrointestinal Disorders	25 (8.65)	1.45 (0.96–2.21)	1.40 (3.09)	0.45 (−0.15)	1.40 (0.92)
Investigations △	20 (6.92)	4.68 (2.95–7.43)	4.32 (52.18)	1.89 (1.23)	4.32 (2.72)
Infections and Infestations	17 (5.88)	1.78 (1.08–2.92)	1.71 (5.28)	0.71 (0.00)	1.71 (1.04)
Metabolism and Nutrition Disorders△	13 (4.5)	4.42 (2.52–7.76)	4.20 (32.22)	1.77 (0.97)	4.20 (2.40)
Skin and Subcutaneous Tissue Disorders	13 (4.5)	1.57 (0.90–2.75)	1.53 (2.52)	0.56 (−0.24)	1.53 (0.87)
Vascular Disorders	12 (4.15)	3.73 (2.08–6.68)	3.56 (22.51)	1.57 (0.74)	3.56 (1.99)
Eye Disorders	9 (3.11)	3.49 (1.79–6.81)	3.38 (15.28)	1.44 (0.51)	3.38 (1.73)
Injury, Poisoning and Procedural Complications	8 (2.77)	0.58 (0.29–1.18)	0.60 (2.28)	−0.68 (−1.66)	0.60 (0.30)
Nervous System Disorders	7 (2.42)	1.02 (0.48–2.18)	1.02 (0.00)	0.02 (−1.02)	1.02 (0.48)
Renal and Urinary Disorders	7 (2.42)	1.96 (0.92–4.17)	1.93 (3.19)	0.78 (−0.26)	1.93 (0.91)
Neoplasms Benign, Malignant and Unspecified (Incl Cysts and Polyps) △	5 (1.73)	27.96 (11.5–67.98)	27.3 (126.56)	2.34 (1.14)	27.25 (11.21)
Cardiac Disorders	3 (1.04)	2.77 (0.89–8.67)	2.75 (3.35)	0.93 (−0.53)	2.75 (0.88)
Musculoskeletal and Connective Tissue Disorders	3 (1.04)	0.51 (0.16–1.59)	0.51 (1.42)	−0.78 (−2.24)	0.51 (0.16)
Ear and Labyrinth Disorders	2 (0.69)	—	—	1.03 (−0.65)	4.29 (1.06)
Product Issues	2 (0.69)	—	—	1.24 (−0.44)	7.44 (1.85)
Psychiatric Disorders	1 (0.35)	—	—	−0.23 (−2.28)	0.75 (0.10)

△, Indicates statistically significant signals in all four algorithms; CI, confidence interval; N, number of reports; IC025, lower limit of the 95% CI, of IC; EBGM05, lower limit of the 95% CI, of EBGM.

The significant AEs meeting the criteria for all four algorithms involved four SOCs: “surgical and medical procedures” (70 reports), “investigations” (20), “metabolism and nutrition disorders” (13), and “neoplasms benign, malignant, and unspecified (including cysts and polyps)” (five).

**FIGURE 2:**
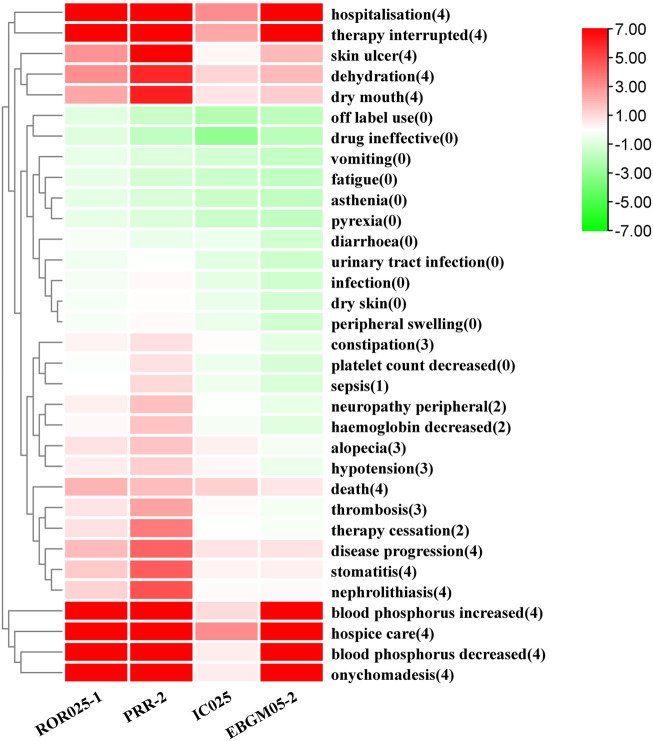
Pemigatinib signal strength at the PT level in the FAERS database The number in parentheses represents the number of methods for which a PT had a signal. ROR, PRR, and EBGM were corrected using 0 as the standard.

Thirty-three PTs corresponding to 11 SOCs reported more than three times were tested for signals of pemigatinib-induced AEs (Table 5; Figure 2). In our study, the most prevalent AEs associated with pemigatinib use were hospitalization (35), death (34), therapy interruption (17), hospice care (16), diarrhoea (9), dehydration (8), alopecia (7), constipation (6), disease progression (6), fatigue (6), and hypotension (6). Thirteen signals of pemigatinib-induced AEs in seven SOCs conformed to the four algorithms simultaneously. The significant PTs of hospitalization, death, therapy interruption, hospice care, dehydration, disease progression, xerostomia, stomatitis, onychomadesis, skin ulcers, increase in the phosphorous level in blood, and decrease in the phosphorous level in blood, all of which are listed on the drug label, were observed. An unexpected significant AE that was not described on the drug label was found in our data-mining: nephrolithiasis.

**TABLE 5:** Pemigatinib signal strength at the PT level in the FAERS database.

System organ class (SOC)	Preferred terms (PTs)	N (%)	ROR (95%two-sided CI)	PRR (χ2)	IC (IC025)	EBGM (EBGM05)
General Disorders and Administration Site Conditions	death △	34 (14.91)	4.40 (3.04–6.36)	3.83 (74.37)	1.82 (1.29)	3.83 (2.65)
	disease progression△	6 (2.63)	6.46 (2.87–14.56)	6.30 (26.85)	1.84 (0.73)	6.30 (2.79)
	fatigue	6 (2.63)	0.79 (0.35–1.78)	0.80 (0.33)	−0.29 (−1.40)	0.8 (0.35)
	drug ineffective	4 (1.75)	0.25 (0.09–0.66)	0.26 (9.00)	−1.71 (−3.02)	0.26 (0.10)
	peripheral swelling	4 (1.75)	2.11 (0.78–5.68)	2.09 (2.29)	0.77 (−0.53)	2.09 (0.78)
	asthenia	3 (1.32)	0.96 (0.31–2.99)	0.96 (0.01)	−0.05 (−1.51)	0.96 (0.31)
	pyrexia	3 (1.32)	0.99 (0.32–3.09)	0.99 (0.00)	−0.02 (−1.47)	0.99 (0.32)
Surgical and medical procedures	hospitalization △	35 (15.35)	23.00 (15.97–33.11)	19.20 (608.66)	3.67 (3.15)	19.18 (13.32)
	therapy interrupted△	17 (7.46)	18 (10.95–29.59)	16.58 (249.88)	3.15 (2.44)	16.56 (10.08)
	hospice care △	16 (7.02)	108.66 (65.11–181.33)	100.17 (1561.61)	3.87 (3.14)	99.51 (59.63)
	therapy cessation	3 (1.32)	5.69 (1.82–17.78)	5.62 (11.41)	1.38 (−0.08)	5.61 (1.80)
Gastrointestinal disorders	diarrhoea	9 (3.95)	1.44 (0.74–2.81)	1.42 (1.16)	0.44 (−0.49)	1.42 (0.73)
	constipation	6 (2.63)	2.92 (1.29–6.57)	2.86 (7.33)	1.17 (0.06)	2.86 (1.27)
	dry mouth△	5 (2.19)	8.32 (3.42–20.21)	8.14 (31.39)	1.89 (0.70)	8.14 (3.35)
	stomatitis△	4 (1.75)	6.53 (2.43–17.58)	6.42 (18.37)	1.62 (0.31)	6.42 (2.39)
	vomiting	4 (1.75)	1.06 (0.39–2.85)	1.06 (0.01)	0.06 (−1.25)	1.06 (0.39)
Infections and infestations	infection	3 (1.32)	2.16 (0.69–6.75)	2.14 (1.84)	0.73 (−0.72)	2.14 (0.69)
	sepsis	3 (1.32)	3.09 (0.99–9.67)	3.06 (4.18)	1.01 (−0.45)	3.06 (0.98)
	urinary tract infection	3 (1.32)	1.94 (0.62–6.06)	1.92 (1.34)	0.64 (−0.82)	1.92 (0.62)
Skin and Subcutaneous Tissue Disorders	alopecia	7 (3.07)	3.73 (1.75–7.92)	3.63 (13.48)	1.45 (0.41)	3.63 (1.71)
	dry skin	4 (1.75)	2.08 (0.77–5.60)	2.06 (2.21)	0.76 (−0.55)	2.06 (0.77)
	onychomadesis△	3 (1.32)	116.53 (37.11–365.97)	114.82 (335.94)	1.96 (0.50)	113.95 (36.28)
	skin ulcer△	3 (1.32)	12.37 (3.95–38.7)	12.2 (30.87)	1.68 (0.22)	12.19 (3.90)
Investigations	blood phosphorus increased△	4 (1.75)	201.29 (74.33–545.11)	197.35 (771.24)	2.29 (0.98)	194.77 (71.92)
	blood phosphorus decreased△	3 (1.32)	89.71 (28.59–281.45)	88.4 (257.73)	1.95 (0.49)	87.88 (28.01)
	haemoglobin decreased	3 (1.32)	3.68 (1.18–11.5)	3.64 (5.76)	1.13 (−0.33)	3.64 (1.16)
	platelet count decreased	3 (1.32)	2.85 (0.91–8.91)	2.82 (3.55)	0.95 (−0.51)	2.82 (0.90)
Vascular Disorders	thrombosis	4 (1.75)	4.61 (1.71–12.41)	4.54 (11.09)	1.41 (0.10)	4.54 (1.69)
	hypotension	6 (2.63)	3.38 (1.50–7.61)	3.31 (9.75)	1.31 (0.20)	3.31 (1.47)
Injury, Poisoning and Procedural Complications	off label use	5 (2.19)	0.47 (0.20–1.15)	0.49 (2.85)	−0.92 (−2.11)	0.49 (0.20)
Metabolism and Nutrition Disorders△	dehydration△	8 (3.51)	8.23 (4.06–16.69)	7.94 (48.75)	2.16 (1.18)	7.94 (3.91)
Nervous System Disorders	neuropathy peripheral	4 (1.75)	3.8 (1.41–10.22)	3.74 (8.08)	1.27 (−0.04)	3.74 (1.39)
Renal And Urinary Disorders	nephrolithiasis△	3 (1.32)	6.86 (2.19–21.46)	6.77 (14.79)	1.47 (0.10)	6.77 (2.16)

△, Indicates statistically significant signals in all four algorithms; CI, confidence interval; N, number of reports; IC025, lower limit of the 95% CI, of IC; EBGM05, lower limit of the 95% CI, of EBGM.

### Comparison between pemigatinib and platinum agents

**FIGURE 3:**
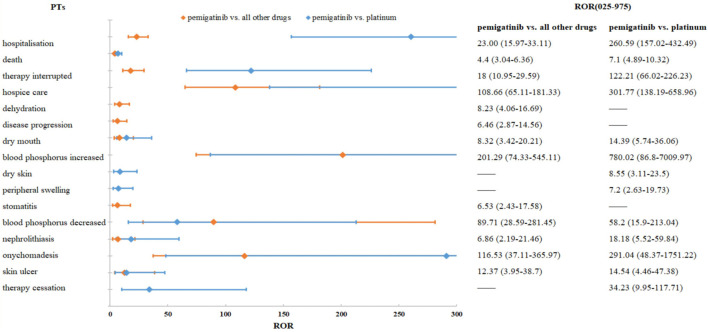
Comparisons of pemigatinib and all other drugs and pemigatinib and platinum agents regarding ROR.

The results of a comparison between pemigatinib and platinum agents in terms of 33 PTs are presented in Table 6. Thirteen PTs met the criteria for all four algorithms: hospitalization, death, therapy interruption, hospice care, xerostomia, dry skin, peripheral swelling, nephrolithiasis, onychomadesis, skin ulcer, therapy cessation, increase in the phosphorous level in blood, and decrease in the phosphorous level in blood. Excluding the latter, the signal values of other PTs were higher for pemigatinib than for other drugs (Figure 3).

**TABLE 6:** Disproportionality analysis of pemigatinib compared with platinum-based therapies.

System organ class (SOC)	Preferred terms (PTs)	N	ROR (95%two-sided CI)	PRR (χ2)	IC (IC025)	EBGM (EBGM05)
General Disorders and Administration Site Conditions	death^+^	34	7.10 (4.89–10.32)	6.08 (143.94)	2.37 (1.84)	5.92 (4.08)
	disease progression	6	0.76 (0.34–1.71)	0.77 (0.44)	−0.34 (−1.45)	0.77 (0.34)
	fatigue	6	0.89 (0.39–2.00)	0.89 (0.08)	−0.15 (−1.26)	0.89 (0.40)
	drug ineffective	4	0.62 (0.23–1.66)	0.62 (0.94)	−0.57 (−1.88)	0.62 (0.23)
	peripheral swelling^+^	4	7.20 (2.63–19.73)	7.08 (20.20)	1.65 (0.32)	6.86 (2.51)
	asthenia	3	0.52 (0.17–1.64)	0.53 (1.27)	−0.74 (−2.20)	0.53 (0.17)
	pyrexia	3	0.35 (0.11–1.11)	0.36 (3.48)	−1.21 (−2.67)	0.36 (0.12)
Surgical And Medical Procedures	hospitalization^+^	35	260.59 (157.02–432.49)	215.83 (3521.34)	4.74 (4.11)	101.91 (61.4)
	therapy interrupted^+^	17	122.21 (66.02–226.23)	112.06 (1181.08)	3.85 (3.03)	71.02 (38.37)
	hospice care^+^	16	301.77(138.19–658.96)	278.06 (1800.78)	3.89 (2.98)	113.88 (52.15)
	therapy cessation^+^	3	34.23 (9.95–117.71)	33.74 (81.04)	1.85 (0.26)	28.83 (8.38)
Gastrointestinal disorders	diarrhoea	9	0.76 (0.39–1.49)	0.77 (0.63)	−0.34 (−1.28)	0.77 (0.40)
	constipation	6	2.06 (0.91–4.66)	2.03 (3.13)	0.81 (−0.31)	2.02 (0.89)
	dry mouth^+^	5	14.39 (5.74–36.06)	14.06 (56.59)	2.11 (0.87)	13.16 (5.25)
	stomatitis	4	2.25 (0.83–6.08)	2.22 (2.68)	0.82 (−0.49)	2.21 (0.82)
	vomiting	4	0.44 (0.16–1.19)	0.45 (2.79)	−0.98 (−2.29)	0.45 (0.17)
Infections and infestations	infection	3	1.05 (0.33–3.29)	1.05 (0.01)	0.04 (−1.42)	1.05 (0.33)
	sepsis	3	0.67 (0.21–2.09)	0.67 (0.49)	−0.45 (−1.91)	0.67 (0.22)
	urinary tract infection	3	2.22 (0.70–6.97)	2.20 (1.95)	0.75 (−0.72)	2.18 (0.69)
Skin and Subcutaneous Tissue Disorders	alopecia	7	1.45 (0.68–3.08)	1.43 (0.92)	0.43 (−0.61)	1.43 (0.67)
	dry skin^+^	4	8.55 (3.11–23.5)	8.40 (25.05)	1.74 (0.40)	8.09 (2.94)
	onychomadesis^+^	3	291.04(48.37–1751.22)	286.75 (341.75)	1.96 (0.08)	115.3 (19.16)
	skin ulcer^+^	3	14.54 (4.46–47.38)	14.34 (34.66)	1.70 (0.18)	13.41 (4.11)
Investigations	blood phosphorus increased^+^	4	780.02 (86.80–7009.97)	764.67 (610.22)	2.28 (0.52)	153.73 (17.11)
	blood phosphorus decreased^+^	3	58.20 (15.9–213.04)	57.35 (127.81)	1.90 (0.25)	44.35 (12.11)
	haemoglobin decreased	3	1.77 (0.56–5.57)	1.76 (0.98)	0.55 (−0.91)	1.75 (0.56)
	platelet count decreased	3	0.66 (0.21–2.08)	0.67 (0.50)	−0.46 (−1.92)	0.67 (0.21)
Vascular Disorders	thrombosis	4	4.98 (1.83–13.57)	4.90 (12.16)	1.44 (0.12)	4.80 (1.76)
	hypotension	6	1.85 (0.82–4.19)	1.83 (2.27)	0.70 (−0.42)	1.82 (0.81)
Injury, Poisoning and Procedural Complications	off label use	5	0.28 (0.12–0.68)	0.30 (9.01)	−1.57 (−2.77)	0.30 (0.12)
Metabolism and Nutrition Disorders	dehydration	8	3.81 (1.87–7.79)	3.70 (15.65)	1.49 (0.50)	3.65 (1.79)
Nervous System Disorders	neuropathy peripheral	4	0.55 (0.20–1.49)	0.56 (1.42)	−0.71 (−2.01)	0.56 (0.21)
Renal And Urinary Disorders	nephrolithiasis^+^	3	18.18 (5.52–59.84)	17.92 (43.86)	1.75 (0.22)	16.47 (5.00)

+, Indicates statistically significant signals in all four algorithms; CI, confidence interval; N, number of reports; IC025, lower limit of the 95% CI, of IC; EBGM05, lower limit of the 95% CI, of EBGM.

### Comparison between pemigatinib and infigratinib

The results of a comparison between pemigatinib and infigratinib in terms of 33 PTs are presented in Table 7. None of the 33 PTs had significant signals in each algorithm method.

**TABLE 7:** Disproportionality analysis of pemigatinib compared with infigratinib^△^.

System organ class (SOC)	Preferred terms (PTs)	N	ROR (95%two-sided CI)	PRR (χ2)	IC (IC025)	EBGM (EBGM05)
General Disorders and Administration Site Conditions	death	34	1.86 (0.62–5.57)	1.72 (1.27)	0.08 (−0.54)	0.6 (0.14)
	disease progression	6	—	—	—	—
	fatigue	6	0.09 (0.03–0.28)	0.12 (25.58)	−1.10 (−2.37)	0.65 (0.21)
	drug ineffective	4	0.25 (0.05–1.18)	0.27 (3.5)	−0.60 (−2.22)	0.69 (0.15)
	peripheral swelling	4	—	—	—	—
	asthenia	3	—	—	—	—
	pyrexia	3	—	—	—	—
Surgical And Medical Procedures	hospitalization	35	—	—	—	—
	therapy interrupted	17	—	—	—	—
	hospice care	16	—	—	—	—
	therapy cessation	3	—	—	—	—
Gastrointestinal disorders	diarrhoea	9	0.33 (0.11–1.05)	0.36 (3.80)	−0.42 (−1.57)	1.08 (0.36)
	constipation	6	0.39 (0.09–1.61)	0.40 (1.83)	−0.40 (−1.80)	0.75 (0.17)
	dry mouth	5	0.32 (0.07–1.40)	0.34 (2.53)	−0.49 (−1.98)	0.69 (0.15)
	stomatitis	4	0.12 (0.03–0.44)	0.13 (13.92)	−1.01 (−2.54)	0.77 (0.24)
	vomiting	4	0.19 (0.04–0.78)	0.2 (6.52)	−0.75 (−2.34)	0.87 (0.22)
Infections and infestations	infection	3	—	—	—	—
	sepsis	3	—	—	—	—
	urinary tract infection	3	—	—	—	—
Skin and Subcutaneous Tissue Disorders	alopecia	7	0.21 (0.07–0.66)	0.24 (8.46)	−0.65 (−1.9)	0.48 (0.13)
	dry skin	4	0.25 (0.05–1.18)	0.27 (3.50)	−0.60 (−2.22)	0.00 (0.00)
	onychomadesis	3	—	—	—	—
	skin ulcer	3	—	—	—	—
Investigations	blood phosphorus increased	4	0.19 (0.04–0.78)	0.20 (6.52)	−0.75 (−2.34)	0.60 (0.14)
	blood phosphorus decreased	3	—	—	—	—
	haemoglobin decreased	3	—	—	—	—
	platelet count decreased	3	—	—	—	—
Vascular Disorders	thrombosis	4	—	—	—	—
	hypotension	6	—	—	—	—
Injury, Poisoning and Procedural Complications	off label use	5	—	—	—	—
Metabolism and Nutrition Disorders	dehydration	8	0.52 (0.13–2.05)	0.54 (0.90)	−0.27 (−1.52)	0.80 (0.19)
Nervous System Disorders	neuropathy peripheral	4	—	—	—	—
Renal And Urinary Disorders	nephrolithiasis	3	—	—	—	—

△, all PTs, have no signal.

## Discussion

Pemigatinib represents considerable advancement in the treatment of CCA with FGFR2 fusions/rearrangements. The AEs associated with pemigatinib are similar to those of other FGFR inhibitors. However, the specific risk of developing AEs after pemigatinib treatment has not been determined precisely.

We investigated the AEs associated with pemigatinib use reported as part of its post-marketing safety assessment. This is the first systematic post-marketing pharmacovigilance investigation of pemigatinib-associated AEs based on the FAERS. We have provided the most thorough and accurate description and characterization of pemigatinib-related AEs to date.

From January 2020 to June 2022, 203 reports of pemigatinib as the prime-suspect medication were documented in the FAERS after the exclusion of duplicate data. Pemigatinib has been on the market for only 2 years and few patients are eligible for such treatment. Hence, the number of AEs related to pemigatinib use reported in the FAERS was small (though the number has grown steadily since the drug was launched). In our study, most AEs reported were from the United States (92.12%), which aligns with the fact that pemigatinib was developed by the American company Incyte and was first marketed in the United States (U S Food and Drug Administration, 2022c). Of the 203 events reported, the main treatment indication was CCA (81.77%), consistent with the indications in the initial drug label. The second most common indication was myeloid/lymphoid neoplasms, a new indication for pemigatinib approved by the FDA on 26 August 2022 (U.S. Food and Drug Administration, 2022a).

Pemigatinib monotherapy was the main regimen (98.52%), and combination therapy was used rarely, consistent with the treatment regimen recommended by guidelines set by the National Comprehensive Cancer Network (National Comprehensive Cancer Network, 2021). Most reports did not provide information on patient age (82.76%), so age-based analyses were not possible.

The expected PTs of the signals detected by the four algorithms simultaneously were hospitalization, death, therapy interruption, hospice care, dehydration, disease progression, xerostomia, stomatitis, onychomadesis, skin ulcers, decrease in the phosphorus in blood, and increase in the phosphorus in blood. These PTs can be summarized into two aspects: abnormal phosphorus level in blood and dermatological AEs.

### Abnormal phosphorus level in blood

Most patients suffering from cancer treated with FGFR inhibitors (e.g., pemigatinib, erdafitinib, infigratinib) develop hyperphosphatemia, which is an undesirable on-target effect (Loriot et al., 2019; Lyou et al., 2020). Hyperphosphatemia is attributable to the inhibition of FGF23 signaling. In health, FGF23 maintains the systemic level of phosphate by stimulating urinary excretion of phosphate (Perwad et al., 2007; Degirolamo et al., 2016). FGFR1 is the primary receptor for the hypophosphatemic effect of FGF23 *in vivo* (Gattineni et al., 2009). The phosphate-lowering functions of FGF23 (which include inhibiting phosphate absorption in the intestine and reducing phosphate reabsorption in proximal renal tubules) can be compromised if FGFR inhibitors interfere with interactions between FGFR1 and FGFR23, which can lead to hyperphosphatemia (Xie et al., 2020; Goyal et al., 2021; Lacouture et al., 2021).

An abnormal phosphorus level in blood was detected in the present study. An increase in the phosphorus level in blood (ROR = 201.29 [95% confidence interval (CI) = 74.33–545.11], PRR = 197.35 [χ^2^ = 771.24], IC = 2.29 [lower limit of the 95%CI of IC (IC025) = 0.98], EBGM = 194.77 [lower limit of the 95%CI of EBGM (EBGM05) = 71.92]) and a decrease in the phosphorus level in blood (ROR = 89.71 [95%CI = 28.59–281.45), PRR = 88.4 [χ^2^ = 257.73], IC = 1.95 [IC025 = 0.49], EBGM = 87.88 [EBGM05 = 28.01]) had strong signals in all four algorithms. In a phase-II study (FIGHT-202) of pemigatinib, the most frequent all-grade AE regardless of cause was hyperphosphatemia (60%, 88/146 patients). In total, 93 (64%) patients experienced an AE of any cause of grade ≥3. Hyperphosphatemia has been reported in 55%–81% of patients with CCA and FGFR rearrangement (Javle et al., 2018; Abou-Alfa et al., 2020; Goyal et al., 2020).

The median time to hyperphosphatemia following treatment initiation was 15 days. This event was treated with a low-phosphate diet, concurrent use of phosphate binders, diuretics, dose reduction, or dose interruption. The highest increase in the serum concentration of phosphate from baseline following pemigatinib treatment was notable in *post hoc* analyses and linked with exposure, thereby illustrating that the phosphate concentration could be used as a proxy for exposure. Hypophosphatemia may be caused by dephosphorization therapy. In one clinical trial, hypophosphatemia occurred in 33 of 146 patients (23%), thereby representing the most common AE of grade ≥3 (Abou-Alfa et al., 2020).

### Dermatological AEs

In addition to on-target AEs, FGFR inhibition can also result in off-target effects affecting the skin. This is a unique class effect of these agents, and includes alopecia, nail changes, and other dermatological events (Touat et al., 2015; Katoh, 2016). These are usually mild-to-moderate in severity.

The pathophysiological processes behind these effects are incompletely understood. Inhibition of FGFRs in keratinocytes, which induces dysregulation of hair-follicle homeostasis and epidermal proliferation and/or differentiation with decreased expression of tight-junction genes in FGFR-deficient mice, are some of the potential mechanisms (J. Yang et al., 2010). Inhibition of hormonal (nonpathological) FGF signaling by FGF19, FGF21, and FGF23 could also be involved (Dieci et al., 2013). The skin toxicity of pemigatinib is connected to the role of FGFR in the division and proliferation of cells (Vogel et al., 2019; Abou-Alfa et al., 2020; Lacouture et al., 2021; Trudeau et al., 2021).

The dermatological AEs detected in our study were onychomadesis (ROR = 116.53 [95%CI = 37.11–365.97], PRR = 114.82 [χ^2^ = 335.94], IC = 1.96 [IC025 = 0.5], EBGM = 113.95 [EBGM05 = 36.28]), skin ulcers (ROR = 12.37 [95%CI = 3.95–38.7], PRR = 12.2 [χ^2^ = 30.87], IC = 1.68 [IC025 = 0.22], EBGM = 12.19 [EBGM05 = 3.9]), xerostomia (ROR = 8.32 [95%CI = 3.42–20.21], PRR = 8.14 [χ^2^ = 31.39], IC = 1.89 [IC025 = 0.7], EBGM = 8.14 [EBGM05 = 3.35]), and stomatitis (ROR = 6.53 [95%CI = 2.43–17.58], PRR = 6.42 [χ^2^ = 18.37], IC = 1.62 [IC025 = 0.31], EBGM = 6.42 [EBGM05 = 2.39]).

### Onychomadesis

The signal value of onychomadesis was highest among skin AEs. Among additional clinically significant AEs, 42% (62/146) of patients reported onychomadesis, which had a median onset of 6.0 months. Because of onychomadesis, 3% (5/146) of individuals required dose reduction, whereas 4% (6/146) required dose interruption (Abou-Alfa et al., 2020).

Onychomadesis was reported in FIGHT-202, but only two reports about this event in routine clinical practice have been published. One patient experienced distal onycholysis of all fingernails. The toenails of another patient fell off because of substantial alterations in her fingernails and toenails (Trudeau et al., 2021; Hoyos et al., 2022).

Often, side-effects related to nails are mild-to-moderate in severity, and usually appear within 1–2 months after treatment initiation. Fewer than 5% of these events necessitate dose reduction/interruption, but this unfavorable effect could affect quality of life and result in skin infections (Hoyos et al., 2022).

### Skin ulcers

Nonuremic calciphylaxis (also termed “intimal vascular calcification”) causes significant skin necrosis, cutaneous ulcerations of grade 3–4, and vascular thrombosis. Changes in the underlying serum phosphatase to be connected to the substance could be responsible for these skin symptoms (Chae et al., 2017). Alternatively, it has been suggested that skin ulcers are related to the role of FGF/FGFR signaling in skeletal development (Su et al., 2014). In addition, skin ulcers can be caused by palmar–plantar erythrodysesthesia syndrome, which has been reported in patients treated with chemotherapy and tyrosine-kinase inhibitors.

### Xerostomia and stomatitis

FGFs and FGFRs are crucial for the branching of salivary glands, and disrupting these factors (or their receptors) influences salivary-gland function (Prochazkova et al., 2018). Patients treated with FGFR inhibitors frequently experience xerostomia (typically grade 1 or 2), and this was the case in 23%–59% of patients with CCA (Lacouture et al., 2021). Xerostomia can be associated with dysgeusia, which can be extremely severe (Lacouture and Sibaud, 2018).

Stomatitis is one of the most commonly observed AEs in patients treated with FGFR inhibitors, with lesions developing quickly after treatment initiation. Stomatitis is characterized by painful, clearly defined lesions, as opposed to oral mucositis caused by radiotherapy or cytotoxic therapy. The prevalence of stomatitis among patients with CCA treated with pemigatinib has been reported to be ≤ 32% (Abou-Alfa et al., 2020). Stomatitis can be excruciatingly painful, and it can lower quality of life markedly even though the condition is typically self-limiting.

### Unexpected PT: nephrolithiasis

All FGFR inhibitors can cause hyperphosphatemia, which is a pharmacodynamic effect of this class of drugs (Gile et al., 2021). Pemigatinib alters phosphate homeostasis, which is important for the development of renal stones or diseases associated with bone loss (Prié et al., 2004). The risk of renal lithiasis or soft-tissue calcification can be increased by an altered phosphate concentration in serum and bone mineralization (Prié et al., 2009).

According to extensive research by Walker and colleagues, stone-formers usually have phosphaturia. Phosphaturia is linked (but not always correlated) to hypercalciuria, increased level of 1,25-dihydroxyvitamin D (1,25 (OH)2D), and occasionally problems with the function of proximal renal tubules. If phosphaturia is in concert with hypercalciuria, this monogenic abnormality increases the risk of renal calcification or stones. In most cases, increased generation of 1,25 (OH)2D in response to hypophosphatemia can be used to explain hypercalciuria development. Thus, instead of a high phosphate concentration in renal tubular fluid, the effect of phosphaturia on the plasma phosphate level increases the risk of stones. An equivalent reaction could be caused by a reduced serum level of phosphate from other sources (Walker, 2019).

The kidneys regulate the phosphate concentration in extracellular fluid by adjusting the need of the body for the reabsorption of filtered phosphate. To prevent the extra-skeletal precipitation of calcium-phosphate deposits caused by excess phosphate or the metabolic and skeletal effects of phosphate deficits, the phosphate concentration in extracellular fluid must be controlled strictly (Prié et al., 2009; Martin et al., 2012).

An unexpected PT, nephrolithiasis, which is not listed on the drug label, was detected in our study. The signal values were 6.86 for ROR (95%CI = 2.19–21.46), 6.77 for PRR (χ^2^ = 14.79), 1.47 for IC (IC025 = 0.10), and 6.77 for EBGM (EBGM05 = 2.16). Compared with platinum agents, the signal values were significantly higher for pemigatinib for all algorithms (ROR = 18.18 [95%CI = 5.52–59.84], PRR = 17.92 [χ^2^ = 43.86], IC = 1.75 [IC025 = 0.22], EBGM = 16.47 [EGBM05 = 5]). These results indicated that pemigatinib was associated with nephrolithiasis. The association of pemigatinib with kidney stones should be noted by clinicians. In addition to the kidneys, calcification has been reported in the liver, which has been considered to be ectopic calcification resulting from the alteration of calcium and phosphorus metabolism by pemigatinib (Yang et al., 2022).

### Risks of pemigatinib compared with platinum drugs or infigratinib

Platinum-based systemic chemotherapy is first-line treatment in patients with advanced-stage CCA who are ineligible for surgical or locoregional options. Pemigatinib displayed efficacy and safety in a phase-II investigation of patients with previously treated locally advanced/metastatic CCA harboring FGFR2 fusions/rearrangements. Therefore, a comparison between pemigatinib with platinum drugs was conducted at the PT level (Brindley et al., 2021).

Death and xerostomia were reported 31.44- and 13.60-fold more often after pemigatinib therapy than after platinum therapy in the FAERS. Compared with platinum agents, the PTs of pemigatinib-associated AEs matching all four algorithms included dry skin and peripheral swelling, but not dehydration or stomatitis. Based on the ROR signal-detection method, excluding a reduction in the phosphorus level in blood, the other PT signal values were higher in the comparison of pemigatinib and platinum than in the comparison of pemigatinib and all other drugs. These findings indicate that the association of these PTs of pemigatinib with platinum drugs was stronger than that with all other drugs. For pemigatinib with platinum, xerosomia and nephrolithiasis were 1.67- and 2.52-fold more frequent than when pemigatinib was employed with other drugs.

The open-label, randomized, active-controlled, multicenter, global phase-III trial FIGHT-302 (NCT03656536) is comparing the efficacy and safety of pemigatinib with gemcitabine plus cisplatin as first-line treatment of patients with advanced CCA and FGFR2 rearrangements (Bekaii-Saab et al., 2020). The results of FIGHT-302 will determine if pemigatinib can replace traditional systemic chemotherapy as first-line treatment for patients with CCA and FGFR2 fusions in the future. The completion date for the primary endpoint of FIGHT-302 is 31 October 2023.

FGFR inhibitors in clinical development include debio 1347, derazantinib, erdafitinib, futibatinib, infigratinib, and pemigatinib. Infigratinib has also been marketed in the United States and is approved for the same indications as pemigatinib. In the present study, comparison between pemigatinib and infigratinib at the PT level showed no significant signals in each algorithm method, which indicated no difference between the two drugs at the PT level (Goyal et al., 2021).

Notwithstanding the benefits of data-mining and real-world large-sample research, our study had three main limitations. First, the FAERS is a spontaneous reporting system that can collect inaccurate and incomplete (missing data) information from many nations and specialists. Consequently, the quality of reports can vary, which could have caused bias in our study. Second, because of the lack of data on all patients taking pemigatinib, determination of the incidence rate of each AE was not possible. Third, disproportionality analysis evaluates only the signal strength, so it did not quantify risk or establish causation. Thus, our study did not reveal causal associations between the target drug and AEs. Third, in the present study, the number of reports of pemigatinib-related AEs was small, so additional studies with more reports are needed to validate our results. Despite these limitations, our results provide a valuable reference for healthcare professionals to monitor the AEs of pemigatinib.

## Conclusion

In addition to AEs consistent with drug specifications and clinical trials (hospitalization, death, therapy interruption, hospice care, dehydration, disease progression, xerostomia, stomatitis, onychomadesis, skin ulcers, increases/decreases in the phosphorus level in blood), we identified a new AE: nephrolithiasis. Moreover, the differences of AEs between pemigatinib and platinum agents were also compared. The association of most PTs of pemigatinib with platinum drugs was stronger than that with all other drugs.

The AEs of pemigatinib are rarely life-threatening, but they can restrict treatment through dose reduction and potentially cause early termination of treatment. Knowledge of the potential AEs of pemigatinib should enable physicians to inform patients about risks and implement efficient treatment strategies promptly to prevent premature dose reduction or termination while maintaining quality of life and curative effects.

The signals detected by our data-mining method could indicate only a statistical association between drug-target AE reports, but not an inevitable cause-and-effect relationship. More clinical studies and real-world studies are needed to further evaluate and find correlations where cause-and-effect relationships exist, and the conclusions obtained must be validated by additional studies.

## Data Availability

The original contributions presented in the study are included in the article/Supplementary material, further inquiries can be directed to the corresponding author.
